# Geographical Correlation and Genetic Diversity of Newly Emerged Races within the Ug99 Lineage of Stem Rust Pathogen, *Puccinia graminis* f. sp. *tritici*, in Different Wheat-Producing Areas

**DOI:** 10.3390/jof8101041

**Published:** 2022-09-30

**Authors:** Atef Shahin, Yasser S. A. Mazrou, Reda Ibrahim Omara, Gamalat Hermas, Mohamed Gad, Ola Ibrahim Mabrouk, Kamel A. Abd-Elsalam, Yasser Nehela

**Affiliations:** 1 Plant Pathology Research Institute, Agricultural Research Center, Giza 12619, Egypt; 2 Business Administration Department, Community College, King Khalid University, Guraiger, Abha 62529, Saudi Arabia or; 3 Department of Agriculture Economic, Faculty of Agriculture, Tanta University, Tanta 31511, Egypt; 4 Department of Agricultural Botany, Faculty of Agriculture, Tanta University, Tanta 31511, Egypt

**Keywords:** wheat, stem rust, *Sr* genes, race analysis, Ug99, molecular markers

## Abstract

Wheat stem rust caused by *Puccinia graminis* f. sp. *tritici* is one of the most destructive wheat diseases worldwide. Identifying stem rust races in general, Ug99 lineage particularly, and determining resistance genes are critical goals for disease assessment. Thirty wheat varieties and monogenic lines with major stem rust resistance genes (Sr) were examined here over the course of three succeeding seasons from 2020 to 2022. Fourteen stem rust races have been identified in ten African countries, as well as Central and West Asia and North Africa (CWANA) and ten European countries. The Ug99 group (Clade I) included four races (TTKSK, TTKST, TTKTK, and TTKTT) and was reported in five African countries (Egypt, Kenya, Rwanda, Tanzania, and Uganda) and Iran, but none of the European countries. On the other hand, none of the races in Clade III-B (TTRTF) and Clade IV-B (TKTTF and TTTTF) were found in Egypt. Furthermore, Egyptian races were clustered separately from races identified from other countries, and six races were found only in Egypt, including PKSTC, RKTTH, TKTTC, TTTSK, TCKTC, and TKTTH. Races from Kenya, Tanzania, Uganda, Rwanda, and Iran were all closely associated with one another, according to correlation analysis. However, most races identified from other investigated regions, including Eritrea, Spain, Ethiopia, Morocco, Italy, Poland, Kenya, Tanzania, and Uganda, were adversely linked with Egyptian races. The diagnostic 350 bp long PCR fragment linked with virulence to *Sr31*, Clement (*Sr31*), and Brigardier (*Sr31*) was used to identify the TTKSK (Ug99) race. The identification of the regional associations and genetic diversity of newly emerged races within the Ug99 lineage of *P. graminis tritici* in Africa, Asia, and Europe is one of the key goals of this study. It will help plant breeders to develop new resistant lines against the virulent races, especially TTKSK (Ug99) and TTTSK. This helps in ensuring global food security in the context of climate change.

## 1. Introduction

After maize, wheat is the second most commonly grown grain in the world. Many diseases affect wheat growth and yield production. Rust diseases are the most prevalent threats that affect wheat plants, such as wheat stem rust, which significantly affects wheat leaves and grain yield [1,2]. Stem rust, caused by *Puccinia graminis* f. sp. *tritici,* is the most destructive disease that seriously threatens wheat grain production [3,4,5]. Stem rust caused severe problems in Africa, the Middle East, Central and West Asia and North Africa (CWANA), Australia, New Zealand, Europe, and the USA [6].

Ug99 race of *P. graminis* f. sp. *tritici* was detected in Uganda in 1998 for the first time. Based on North American nomenclature [7], the initial race was identified as TTKSK, with pathogenicity to the stem rust resistance gene *Sr31*. It is also virulent to a wide range of wheat resistance genes, as well as others of unknown origin. The Ug99 lineage extended over the eastern African highlands, including Zimbabwe, South Africa, Sudan, Yemen, and Iran. Because 90 percent of known wheat cultivars are susceptible to Ug99 races, it is often regarded as the most serious danger to global wheat production [8]. Resistant wheat varieties are uncommon in Kenya and Ethiopia. New sources of race-specific resistance from various wheat relatives must be found and transmitted to improve resistance. Major efforts are required to replace Ug99 susceptible types with those that have diverse race-specific or long-lasting resistances, thereby reducing the Ug99 threat.

The Ug99 (TTKSK) was reported first in Yemen and then throughout the Middle East and Asia [5]. In March 2008, the Food and Agriculture Organization (FAO) registered Ug99 (race TTKSK) in Iran [9]. Detection in Iran in 2007 was followed by drought conditions, and no reports of Ug99 were received from Iran in 2008. However, in 2009, Ug99 was found in the southern Iranian province of Khuzestan, where spring wheat is grown, and weather conditions are favorable.

The pathogen is changing rapidly; approximately fifteen known variants have been identified within the Ug99 lineage. All are closely related, having nearly identical DNA fingerprints, but they differ slightly in their avirulence/virulence profiles [10]. In contrast, these races are very different from other known races in the United States and South Africa. The similarity of Ug99 races’ simple sequence repeats markers indicates that they evolved from a common ancestor. Pathogen surveillance reveals that known variants in the Ug99 lineage are expanding across Africa. The majority of the substantial differences were identified in Kenya. Notable recent developments include the confirmation of coupled virulence to *Sr31* and *Sr24* (race PTKST) from South African isolates collected in 2009 [11], as well as the same race in Ethiopia from isolates collected in 2007.

New variants are expected to continue spreading, and it is likely that more will be found within the Ug99 race. Currently, race TTKSK is the only known pathotype in the Ug99 lineage that has been reported outside Africa, although the development of more variations outside Africa is thought to be plausible. Reports from various African countries are being verified. TTKST and TTTSK, for example, were reported from Uganda in 2012, TTKSK from Eritrea in 2012, and Egypt and Rwanda in 2014 [12,13]. Moreover, Ug99 (TTKSK) of wheat stem rust fungus was reported in Egypt in 2020 [14]. Additionally, the Ug99 race TTKTT of wheat stem rust was recently reported in Iraq [15].

Airflows out of Yemen followed the same fundamental pattern as in previous years, with air movement from Yemen to southern Iran during the 2009–2010 winter seasons. One minor change in projected airflows from 2009 to 2010 vs. previous years was a tendency for air to travel northwesterly out of Yemen towards Israel and Jordan. In 2010, Israel and Lebanon reported stem rust. However, it is unknown whether Ug99 was present. Given the revised likely migratory paths provided by Singh et al. [5], the presence of Ug99 or its variations in this region may have future implications for Egypt’s wheat fields. In the present research, we continued efforts made by researchers throughout the world to fill in a missing piece of the puzzle regarding the emergence and dissemination of novel races of the wheat stem rust fungus, particularly those belonging to the Ug99 lineage. In order to identify the Ug99 race using a molecular marker, we first sought to determine the geographic relationship and genetic diversity of newly generated races of the stem rust pathogen, *P. graminis tritici*, throughout Africa, Asia, and Europe. In order to confirm the virulence formula of 14 recently discovered races of the stem rust pathogen, we assessed the most prevalent monogenic lines (*Sr* genes).

## 2. Materials and Methods

### 2.1. Evaluation of Thirty Wheat Lines with Stem Rust Resistance Genes at Adult Plant Stage under Field Conditions

Thirty wheat lines with stem rust resistance genes (Sr^’^s) were obtained from the International Maize and Wheat Improvement Center (CIMMYT, México-Veracruz, El Batán, Mexico) and National Small Grain Collection (Aberdeen, ID, USA) and evaluated in two Egyptian governorates, Kafrelsheikh and Sharqia, during three successive growing seasons: 2020, 2021, and 2022 (Table 1). The monogenic lines described in Table 1 were planted in a three-replicate randomized complete block design (RCBD), with each plot consisting of three rows (3 m long and 30 cm apart). Each row received 5 g of the evaluated monogenic line. A spreading area planted with combinations of the extremely sensitive variety, Morocco, encircled the experiment. After the heading stage, disease responses to the stem rust pathogen were recorded as disease severity (percent) was measured in the tested genotypes using the amended Cobb’s scale, as proposed by Peterson et al. [16]. Infection types were recorded according to Roelfs et al. [17], i.e., highly resistant (0), resistant (R), moderately resistant (MR), moderately susceptible (MS), and susceptible (S).

### 2.2. Stem Rust Samples Identified in Egypt

In the 2020–2022 growing seasons in the Kafrelsheikh and Sharqia governorates, a stem rust survey was carried out annually to collect infected stems displaying the typical symptoms from wheat monogenic lines and types with substantial stem rust resistance genes (*Sr*). Paper envelopes (6 × 20 cm) were utilized to gather and store the fifty-seven samples that were obtained. Collected samples (infected stems) were placed on top of their envelopes for the whole night in a room-temperature environment in order to minimize humidity within the samples. After that, samples were maintained until the end of the season in desiccators with calcium chloride at 4 °C in a refrigerator [18].

### 2.3. Stem Rust Samples Send to Global Rust Reference Center (GRRC)

The infected samples were collected from Egypt (Kafrelsheikh and Sharqia Governorates); from 9 Africa and CWANA countries, i.e., Eritrea, Ethiopia, Iran, Kenya, Morocco, Rwanda, Tanzania, Tunisia, Uganda; and from 10 countries in Europe, i.e., Czech Republic, Denmark, France, Germany, Hungary, Italy, Poland, Slovakia, Spain, and Sweden (in total 625 samples; only 23 of them were collected from Egypt) were sent to the Global Rust Reference Center (GRRC) in Denmark (Table 2).

### 2.4. Race Analysis

The highly susceptible variety Morocco (7-day-old seedlings) was inoculated with urediniospores from each sample collection at the stem rust greenhouse located in the Wheat Diseases Department, Plant Pathology Research Institute, Agricultural Research Center, Egypt, and in GRRC in Denmark. The procedure for vaccination was followed as stated by Stakman et al. [18]. To create an initial film of free water on the plants, which is necessary for spores to germinate and for the infection to take hold, the seedling leaves were rubbed gently with tap water between wet fingers, sprayed with water in the incubation chambers, then inoculated by shaking or brushing rusted materials over the plant leaves. Inoculated seedlings were incubated in a secured glass moist chamber in darkness for 16 h at 18–22 °C and 98–100% relative humidity for 24 h to let the rust spores grow and infect the plants. Subsequently, inoculated plants were transferred to be maintained in the greenhouse at 25–28 °C, with 80–90% relative humidity, 16 K Lux light intensity, and a 16 h light, 8 h dark photoperiod. Inside the greenhouse, each sample was transferred to a place designated for it, covered with an acrylic cylinder so that there was no mixing between the samples, and watched until rust pustules appear. To obtain enough urediniospores to inoculate the differential sets, three single pustules from each sample were separated individually before being inoculated onto the seedling of a highly susceptible wheat variety from Morocco. Each pustule infected a single plant (20 *Sr^,^s*).

The plant response was determined on 20 lines divided into 5 groups of 4 lines each, as described by Njau et al. [7], with slight modifications. The first group consists of isogenic *Sr*-lines 5, 21, 9e, 7b; the second group consists of 11, 6, 8a, 9g; the third group consists of 36, 9b, 30, 17; the fourth group consists of 9a, 9d, 10, Tmp; and the fifth group consists of lines 24, 31, 38, McN [20]. Based on the combination of responses from low infection type (L) and high infection type (H) plants, *P. graminis* coding (Pt-code) for each isolate was defined using capital letters (Table 3).

### 2.5. Disease Evaluation

After 12 days of the formation of pustules on near-isogenic lines (20 Sr^’^s), wheat stem rust infection types for all isogenic lines were documented using a standard illness rating scale of 0–4. Stakman et al. [18] revealed the virulence patterns on differential sets based on the low infection types produced by each line in response to infection (infection types 0, 1, and 2 were avirulent, whereas infection types 3 and 4 were virulent).

### 2.6. DNA Extraction and PCR Amplification

DNA was extracted from 0.1g spores collected from pure cultures maintained on susceptible wheat seedlings in our greenhouse using a genomic plant DNA extraction based on Dellaporta et al. [21] method. Three biological replicates per treatment were analyzed. The purity and concentration of the fungal DNA were determined using a NanoDrop 2000 spectrophotometer (Thermo Fisher Scientific, Waltham, MA, USA), and DNA concentration was adjusted to 50 ng μL^−1^ for further PCR amplification. The PCR amplification was performed in a 25 µL reaction mixture using two particular primers (PgtSSR3F: 5′-GGACCAAAACCAGAACCAGA-3′ and PgtSSR3R: 5′-CCCACTCCTAATCCTCACGA-3′) that were designed for Ug99-associated markers [20]. The PCR reaction mixture contained 2.5 µL genomic DNA (50 ng μL^−1^), 1.0 µL of each reverse and forward primers (10 picomols), and 8.0 µL Milli-Q^®^-H_2_O. After optimization, DNA amplification was performed using a thermal cycler (Rocorbett-Research, CG1-96). The thermal cycler was programmed for one cycle of denaturation at 94 °C for 5 min followed by 35 cycles of denaturation at 94 °C for 30 s, annealing at 55 °C for 1 min, and extension at 72 °C for 1 min. A final extension step at 72 °C for 10 min was also performed [22]. PCR products were resolved by electrophoresis in 1.2% agarose gel at 100 V/1 h and visualized under UV light following staining with ethidium bromide (500 µL L^−1^). Mid-Range DNA Ladder 100 bp-3 kbp linear sale (Jena Bioscience, Jena, Germany) was used to determine the molecular weight of the tested materials.

### 2.7. Statistical Analysis

Two-way hierarchical cluster analysis (HCA) was used to better understand the relationship between the 14 newly emerged physiological races of *P. graminis* f. sp. *tritici*, their geographical distribution, and/or their virulence formula. Briefly, the presence/absence of the 14 races of *P. graminis tritici* was converted to a binary data matrix (0 = absence and 1 = presence), as well as the virulence formula of the 14 races of *P. graminis tritici* (0 = avirulent and 1 = virulent) and used for further statistical analysis. Distance and linkage were measured using Ward’s minimum variance method [23], with 95% confidence between groups. Moreover, principal component analysis (PCA) was performed, and its associated scatter plots and loading plots were generated using the singular value decomposition (SVD). Furthermore, correlation analysis was computed to measure the strength of the relationship between newly emerged physiological races and their geographical distribution and their virulence formula.

## 3. Results

### 3.1. Evaluation of the Tested Wheat Lines and Varieties with Sr Genes to Stem Rust Disease at the Adult Stage under Field Conditions

**Figure 1:**
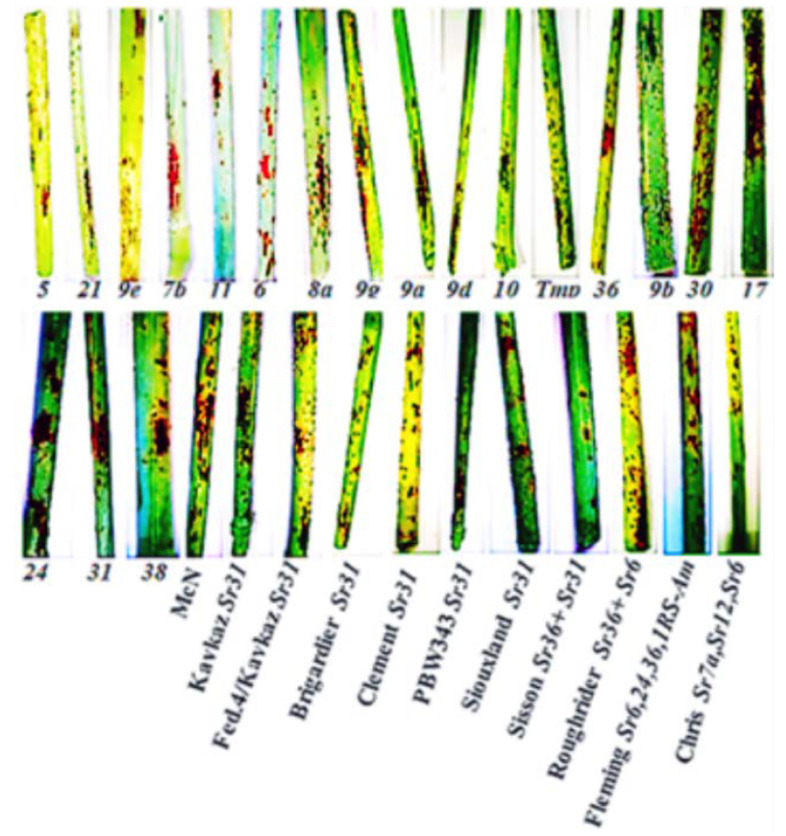
Symptoms of wheat stem rust on 30 wheat lines and varieties with stem rust resistance genes (*Sr*) at Sharqia location during the 2021 growing season.

In three subsequent seasons, at two distinct locations, the responses of 30 lines and varieties with stem rust resistance genes (*Sr,s*) were assessed (Table 4 and Figure 1). At the Kafrelsheikh location, the wheat lines and varieties with *Sr* genes, namely, Kavkaz (*Sr31*), Federation4/Kavkaz (*Sr31*), Brigardier (*Sr31*), PBW343 (*Sr31*), Fleming *(Sr6, 24, 36, 1RS-Am),* and Chris (*Sr7a, Sr12, Sr6*), were resistant to moderate resistance during the three seasons (Table 4). The *Sr24*, *Sr31, Sr38*, Clement (*Sr31*), Siouxland (*24 + 31*), and Sisson (*36 + 31*) wheat lines and varieties were resistant to moderately resistant in seasons 2020 and 2021 and moderately resistant to moderately susceptible in season 2022. Moreover, the line with the *Sr36* gene was susceptible (10S) in the third season (2022). On the other hand, wheat lines and varieties with *Sr* genes; *5, 21, 9e, 7b, 11, 6, 8a*, *9g, 9b, 30, 17, 9a, 9d, 10, Tmp*, and *MCN* were susceptible to stem rust (ranged from 10S to 90S) during the 2020, 2021, and 2022 growing seasons (Table 4).

At the Sharqia location, the disease severity ranged between 5S and 60S with lines with *Sr* genes, namely, *5, 21, 9e, 7b, 11, 6, 8a, 9g, 9b, 36, 9b, 30, 17, 9a, 9d, 10, Tmp,* and *MCN,* during the three seasons (Table 4 and Figure 1). On the other hand, lines with *Sr38* gene, namely, Federation4/Kavkaz (*31*), Brigardier (*31*), Clement (*31*), Siouxland (*24 + 31*), Roughrider (*Sr36 + 6*), Fleming (*Sr6, 24, 36, 1RS-Am*), and Chris (*Sr7a, Sr12, Sr6*), were resistant (0) in the 2020 and 2021 seasons. Moreover, in the third season (2022), line *Sr38* was susceptible (40S), whereas lines such as Federation4/Kavkaz (*Sr31*), Brigardier (*Sr31*), Clement (*Sr31*), Siouxland (*Sr24+ Sr31*), Roughrider (*Sr36+ Sr6*), Fleming (*Sr6, Sr24, Sr36, 1RS-Am*), and Chris (*Sr7a, Sr12, Sr6*) were moderately resistant to susceptible (10MR-MS). Additionally, during the 2021 and 2022 seasons, the *Sr24* gene-carrying line, *Sr31* gene-carrying lines, and Kavkaz (*Sr31*) were all moderately susceptible to susceptible (Table 4 and Figure 1).

### 3.2. Survey of Wheat Stem Rust and Race Identification in Egypt

Infected samples were gathered based on the analysis of 30 wheat monogenic lines and types with stem rust resistance genes over the course of the preceding three successive seasons. The Sharqia governorate had the largest number of successfully isolated stem rust samples (36 samples and 20 isolates over the course of three seasons), followed by the Kafrelsheikh governorate (21 samples and 13 isolates over the course of three seasons). Moreover, the largest number of collected samples and successful races were in 2020 (23 samples and 15 isolates), followed by the 2021 (20 samples and 11 isolates) and the 2022 season (14 samples and 7 isolates), as indicated in Table 5.

**Figure 2:**
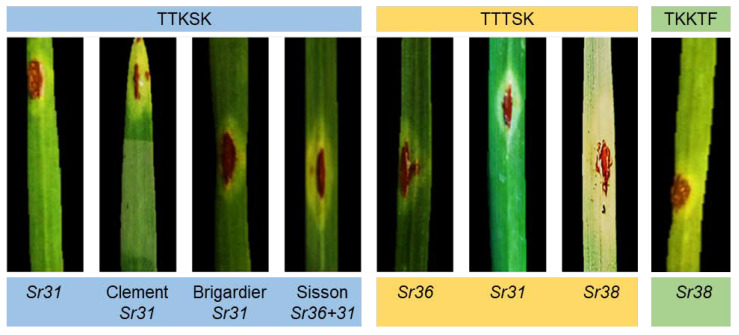
Identified TTKSK, TTTSK, and TKKTF races in the seedling stage and their symptoms on wheat monogenic lines and varieties with stem rust resistance genes.

Eight stem rust races were identified during the three seasons in Kafrelsheikh and Sharqia governorates (Table 6). In 2020, seven races were identified, such as PKSTC, RKTTH, TCKTC, TKKTF, TKTTC, TKTTH, and TTKSK. In 2021, six races were identified, i.e., PKSTC, RKTTH, TKKTF, TKTTC, TTKSK, and TTTSK. The most frequent race was TKTTF (50.0%) in the 2021 and 2022 seasons, respectively. In 2022, four races were identified, such as TCKTC, TKTTH, TTKSK, and TTTSK. The most frequent race was TTKSK (50.0%) in this season. On the other hand, the TTKSK race was identified in all seasons, and the new race TTTSK was only detected in Egypt (Figure 2).

### 3.3. Geographical Distribution of Newly Emerged Races of Puccinia graminis f. sp. tritici in Africa, Asia, and Europe

**Figure 3:**
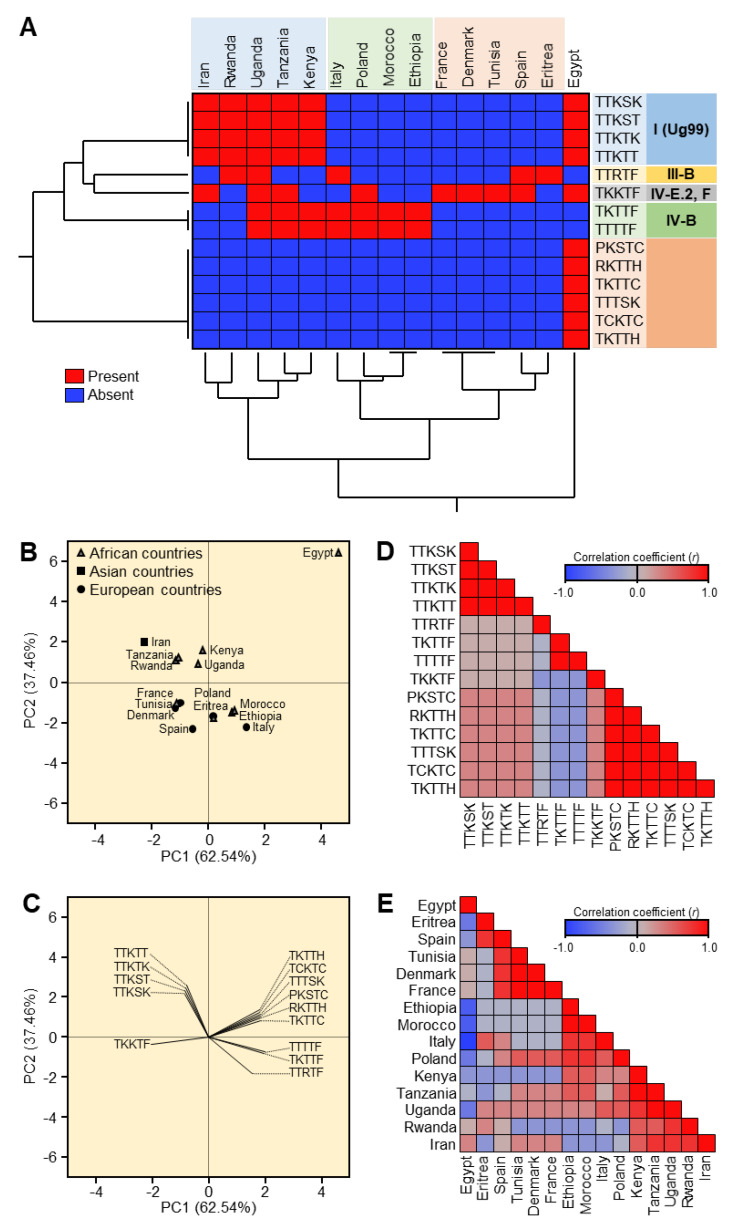
Two-way hierarchical cluster analysis (HCA) and Principal component analysis (PCA) of Geographical distribution of newly emerged races of *Puccinia graminis* f. sp. *tritici* in Africa, Asia, and Europe. (**A**) Two-way HCA is based on the presence/absence of the 14 races of *P. graminis tritici* in 15 different countries in Africa, Asia, and Europe. The heatmap diagram depicts the presence/absence of *P. graminis* f. sp. *tritici* races, with red representing the presence and blue representing the absence. Individual races are represented by rows, while locations/countries are represented by columns. PCA-related scatter plot and loading plot (**B**,**C**). Correlation analysis of different races and their geographical distribution (**D**,**E**). The heat map diagram depicts the correlation coefficient I. Positive correlation coefficienI(*r*) are highlighted in red, whereas negative correlation coefficIts (*r*) are highlighted in blue (see the scale at the upper right corner of the heat map).

The stem rust samples collected from Egypt, Eritrea, Ethiopia, Iran, Kenya, Morocco, Rwanda, Tanzania, Tunisia, Uganda, Czech Republic, Denmark, France, Germany, Hungary, Italy, Poland, Slovakia, Spain, and Sweden were sent to GRRC in Denmark (Table 2). The Ug99 group (Clade I) included four races (TTKSK, TTKST, TTKTK, and TTKTT) and was reported in five African countries (Egypt, Kenya, Rwanda, Tanzania, and Uganda) and Iran but in none of the European countries (Figure 3A). On the other hand, none of the races in Clade III-B (TTRTF) and Clade IV-B (TKTTF and TTTTF) were found in Egypt. Furthermore, HCA showed that Egypt was clustered separately from all other countries, and six races were found only in Egypt, including PKSTC, RKTTH, TKTTC, TTTSK, TCKTC, and TKTTH (Figure 3A).

The presence/absence of the 14 races was used to perform principal component analysis (PCA) in all of the areas investigated. Figure 3B,C illustrate the scatter plot and loading plot related with PCA. The PCA-associated scatter plot clearly distinguished Egypt from all other places, which overlapped in the center of the scatter plot (Figure 3B). It is worth mentioning that PC1 (Eigenvalue = 8.76) and PC2 (Eigenvalue = 5.24) donated 62.54% and 37.46% of the total variance, respectively. Furthermore, the PCA-associated loading plot (Figure 3C) showed that six races, including PKSTC, RKTTH, TKTTC, TTTSK, TCKTC, and TKTTH, were positively correlated with Egypt, whereas other races were correlated with other countries, in agreement with the HCA analysis.

To better understand the relationships between different races and their geographical distribution, correlation analysis was carried out. Briefly, correlation analysis showed that PKSTC, RKTTH, TKTTC, TTTSK, TCKTC, and TKTTH were highly geographically correlated to each other and slightly correlated with TTKSK, TTKST, TTKTK, and TTKTT, but negatively correlated with TTRTF, TKTTF, and TTTTF (Figure 3D). Likewise, Kenya, Tanzania, Uganda, Rwanda, and Iran were highly correlated to each other (Figure 3E). On the other hand, Egypt was negatively correlated with most of the studied locations, such as Eritrea, Spain, Ethiopia, Morocco, Italy, Poland, Kenya, Tanzania, and Uganda, or at least no significant relationship was noticed, such as Tunisia, Denmark, France, and Rwanda (Figure 3E).

**Figure 4:**
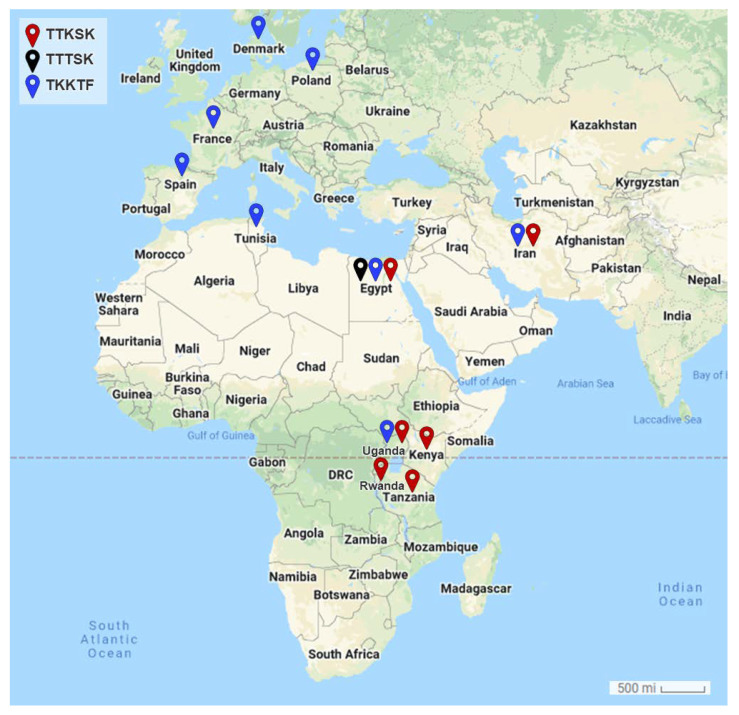
Geographical distribution of the most virulent races; TTKSK, TTTSK, and TKKTF of stem rust pathogen, *Puccinia graminis* f. sp. *tritici* in Africa, Asia, and Europe. Red symbols signify TTKST races, black symbols signify TTTSK races, whereas blue symbols signify TKKTF races, see the key at the top left corner of the map.

The geographical distribution of TTKSK (represents Clade I [Ug99]), TTTSK (represents the races that are only found in Egypt), and TKKTF (represents Clade IV-E.2, F) physiological races of stem rust pathogen, *Puccinia graminis* f. sp. *tritici*, in Africa, Asia, and Europe is shown in Figure 4. While the Ug99 group was mostly reported from African countries, the Clade IV-E.2, F-associated races were mostly reported from Europe, according to the map. It is worth noting that Egypt has been described as a nexus for the three physiological races depicted on the map in Figure 4.

### 3.4. Virulence Formula Refers to the Genetic Diversity between Newly Emerged Races Puccinia graminis tritici

**Figure 5:**
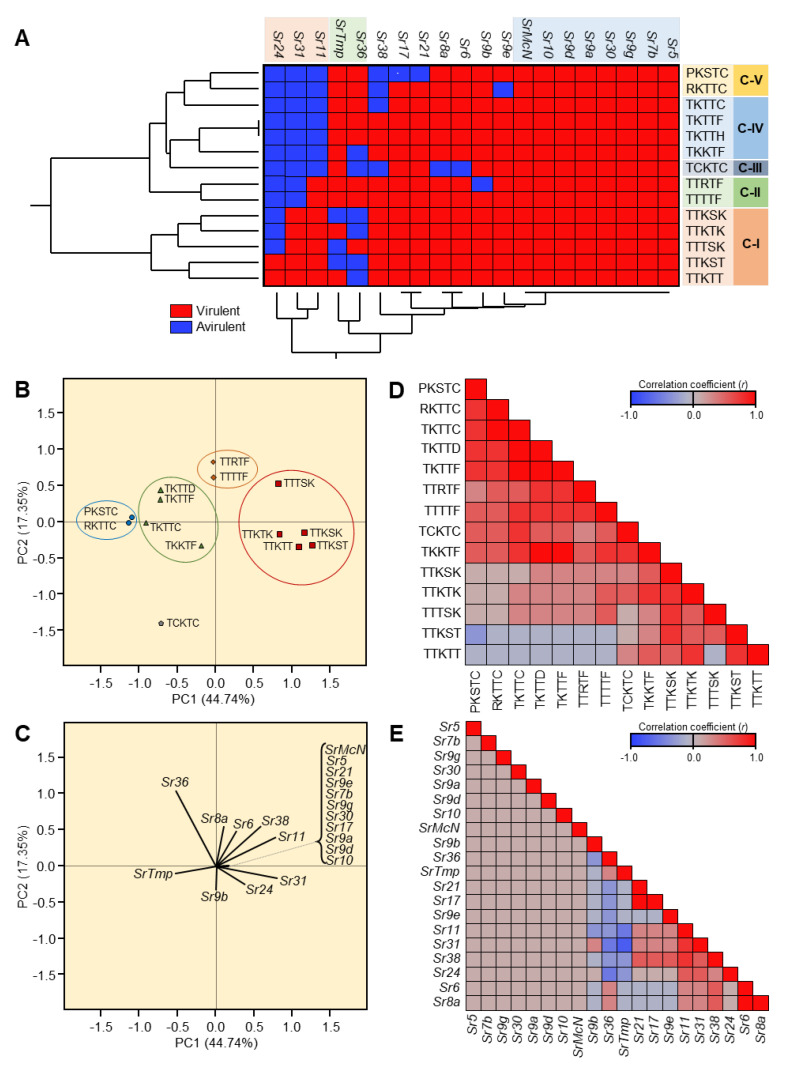
Two-way hierarchical cluster analysis (HCA) and principal component analysis (PCA) of virulence formula of 14 newly emerged races of *Puccinia graminis* f. sp. *tritici* in Africa, Asia, and Europe on 20 stem rust differential lines. (**A**) Two-way HCA is based on the virulence formula of the 14 races of *P. graminis tritici* on 20 stem rust differential lines. The heatmap diagram depicts the virulence of *P. graminis* f. sp. *tritici* races, with red representing virulent reactions and blue representing avirulent reactions. Individual races are represented by rows, whereas monogenic lines are represented by columns. PCA-related scatter plot and loading plot (**B**,**C**). Correlation investigation between different races and 20 stem rust differential lines (**D**,**E**). The heat map diagram depicts the correlation coefficient (r). Positive correlation coefficients (*r*) are highlighted in red, whereas negative correlation coefficients (*r*) are highlighted in blue (see the scale at the upper right corner of the heat map).

Fourteen stem rust races have been identified from Africa, CW Asia, and Europe in stem rust greenhouse in Egypt and GRRC in Denmark (Table 7 and Figure 5). The most virulent race was TTKTT, which was virulent to 19 monogenic lines, followed by TTTSK, TTKST, TTKTK, and TTTTF, which were virulent to 18 monogenic lines (Table 7). On the other hand, races TCKTC and PKSTC were the lowest virulent races, which were virulent to only 13 and 14 monogenic lines, respectively (Table 7 and Figure 5A).

Furthermore, two-way HCA was used to better understand the genetic background of individual physiological races of *P. graminis f. sp. tritici* and their virulence to 20 monogenic lines. Briefly, the HCA showed that the 14 studied races were separated into five distinct clusters. Cluster I (C-I) included the highly virulent races TTKSK, TTKTK, TTTSK, TTKST, and TTKTT, whereas TTRTF (virulent to 17 lines) and TTTTF (virulent to 18 lines) were clustered together in Cluster II (C-II) (Figure 5A). On the other hand, the lowest virulent race, TCKTC, was clustered separately in Cluster III (C-III). Likewise, TKTTC, TKTTF, TKTTH, and TKKTF were clustered together within Cluster IV (C-IV), and PKSTC and RKTTC were clustered together within Cluster V (C-V) (Figure 5A). Moreover, HCA between lines showed that eight lines (*Sr5, Sr7b, Sr9g, Sr30, Sr9a, Sr9d, Sr10,* and *SrMcN*) were susceptible to all studied races and clustered together on the right side of the dendrogram. Line *Sr24* was the most resistant line (resistant to 12 races), followed by line *Sr31* (resistant to 9 races), and line *Sr11* (resistant to 7 races) (Figure 5A).

In agreement with HCA, the PCA-associated scatter plot showed a clear separation between the 14 studied races, which were clustered into five separate groups (Figure 5B). PC1 (Eigenvalue = 0.78) and PC2 (Eigenvalue = 0.30) donated 44.74% and 37.46% of the total variance, respectively. Furthermore, the PCA-associated loading plot (Figure 5C) showed that lines *Sr11*, *Sr31*, and *Sr24* were positively correlated with TTTSK, TTKST, TTKSK, TTKTK, and TTKTT.

To measure the strength of the relationship between the newly emerged races and monogenic lines, correlation analysis was carried out. Correlation analysis between physiological races showed a positive association (ranging from low to high) between most races, except TTKST and TTKTT, which showed a low negative correlation with PKSTC, RKTTC, and TKTTC, TKTTD, TKTTF, TTRTF, and TTTTF (Figure 5D). Likewise, correlation analysis between monogenic lines showed no association between wheat lines and varieties with stem rust resistance genes (Sr), such as lines *Sr5, Sr7b, Sr9g, Sr30, Sr9a, Sr9d, Sr10, and SrMcN,* and all other study lines. However, a moderate-to-high correlation was observed between lines *Sr11, Sr31, Sr38, Sr24,* and *Sr6* (Figure 5E).

### 3.5. PCR-Based DNA Markers

**Figure 6:**
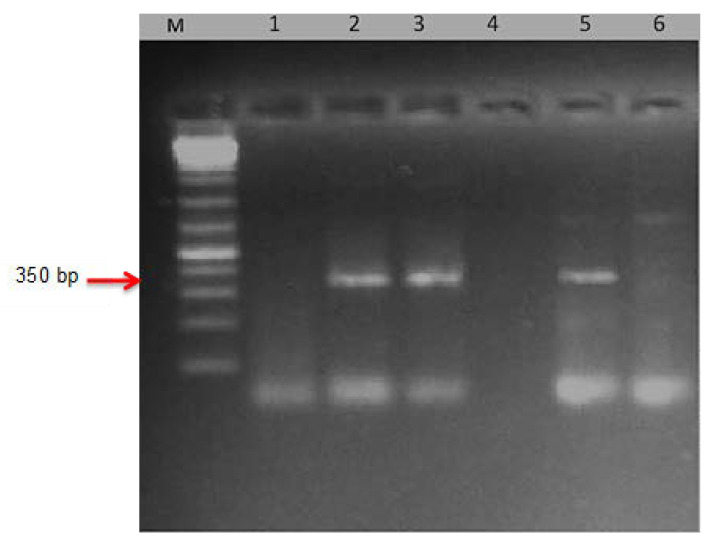
Electrophoretic amplified pattern of DNA extracted from six races of *Puccinia graminis* using the specific primer for *Ug99*. M= DNA Ladder (DNA Marker), Lane 1 = Race isolated from the line with *Sr24* gene; Lane 2 = Race isolated from the line with *Sr31* gene; Lane 3 = Race isolated from Clement (with *Sr31* gene); Lane 4 = Race isolated from the line with *Sr38*; Lane 5 = Race isolated from Brigardier (with *Sr31* gene); and Lane 6 = Race isolated from the line Fleming (with *Sr6, Sr24, Sr36,* and *1RS-Am* genes).

The definition of UG99 (TTKSK) was confirmed by molecular markers using specific primers. Urediniospores were collected from the individual pustules of *Puccinia graminis* on wheat lines and varieties with stem rust resistance genes (*Sr*); lines *Sr24, Sr31*, *Sr38*, Clement (*Sr31*), Brigardier (*Sr31*), and Fleming (*Sr6, 24, 36, 1RS-Am*). Then, the DNA of these samples was isolated and specific primers of Ug99-TTKSK were used. Data illustrated in Figure 6 show that the diagnostic PCR fragment indicating UG99 (TTKSK) race was detected in *P. graminis* f. sp. *tritici* isolates from cvs Clement and Brigardier (with *Sr31* gene) as a fragment 350 bp. On the other hand, lines *Sr24, Sr38,* and Fleming (*Sr6, Sr24, Sr36, 1RS-Am*) were not detected in any fragments with UG99 (TTKSK) at 350 bp. (Figure 6).

## 4. Discussion

Historically, stem rust disease has been a serious threat in several wheat-producing regions [9]. More than 20 years ago, race Ug99 of *P. graminis f. sp. tritici* was first detected in Uganda and identified as TTKSK based on the North American nomenclature [7]. Up to today, approximately 15 variants were identified within the Ug99 lineage. These variants are spreading to several wheat-producing countries in the Eastern African highlands, as well as Zimbabwe, South Africa, Sudan, Yemen, and Iran. Races of the Ug99 lineage pose a threat to wheat productivity and food security [11] due to their capacity to infect almost 90% of the kinds of wheat grown globally. Unfortunately, we know very little regarding the presence of the Ug99 race in Egypt. Although Patpour et al. [13] and Shahin et al. [14] reported the Ug99 race in Egypt, some Egyptian varieties, such as Misr-1 and Misr-2, were chosen from CIMMYT wheat genotypes and tested in Uganda, Kenya, and Ethiopia, and they proved their resistance to stem rust disease, especially for this race [24]. Then, these two varieties were imported for cultivation in these countries to overcome this race (Ug99). As a result, most countries are attempting to speak one language in the field of wheat stem rust disease. As a result, it was critical to use a molecular marker to analyze the geographical connection and genetic diversity of newly generated races of the stem rust pathogen, *P. graminis* f. sp. *tritici*, across Africa, Asia, and Europe, as well as the Ug99 race.

Our findings showed that disease severity of stem rust disease varied from one location to another and from one season to another. For instance, in the Kafrelsheikh governorate, the monogenic lines Kavkaz (*Sr31*), Federation4/Kavkaz (*Sr31*), Brigardier (*Sr31*), PBW343 (*Sr31*), Fleming *(Sr6,24,36,1RS-Am),* and Chris (*Sr7a, Sr12, Sr6*) were resistant during the three seasons under study. On the other hand, wheat lines and varieties with *Sr* genes, namely, lines *Sr24*, *Sr31, Sr38,* Federation4/Kavkaz (*31*), Brigardier (*31*), Clement (*31*), Siouxland (*24 + 31*), Roughrider (*Sr36 + 6*), Fleming (*Sr6, 24, 36, 1RS-Am*), and Chris (*Sr7a, Sr12, Sr6*) were susceptible to stem rust at the Sharqia location in the third season. Accordingly, it can be concluded that the presence of virulent races of *P. graminis* was able to break the resistance of *Sr* genes, which we noticed early in the field.

Previously, Hasan and Abou-Zeid [25] reported that lines *Sr31, Sr24, Sr38,* and *Sr36* were the most resistant genes, and Abebe et al. [26] reported that lines *Sr24* and *SrTmp* were effective for all tested isolates; however, our findings do not support this point of view. Taken together, we suggest that changes in the behavior of some lines, i.e., *Sr24*, *Sr31, Sr36,* and *Sr38*, are responsible for resistance and had to be interpreted, especially with the first identification of the Ug99 [15].

As summarized by Singh et al. [12], Ug99 had been confirmed in Uganda (1998), Kenya (2001), Ethiopia (2003), Sudan (2006), Yemen (2006), Iran (2007), and Tanzania (2009). This pathotype/race can overcome many *Sr* genes in which virulence had not previously been detected. For instance, race Ug99 is the first known race of *P. graminis* f. sp. *tritici* that carries virulence to gene *Sr31* located on 1BL. 1RS translocation from rye (*Secale cereale*) [27] as well as virulent to the majority of *Sr* resistance genes of wheat origin such as gene *Sr38,* introduced into wheat from *Triticum ventricosum* [20]. This strong virulence combination in Ug99 explains why wheat cultivars and germplasm are susceptible all across the world. Because of the breakdown of these critical genes, it is necessary to better understand the geographical association and genetic diversity of newly formed races within the Ug99 lineage of the stem rust pathogen in various wheat-producing locations in general, and Egypt in particular.

Fourteen stem rust races have been identified in Egypt and other countries in the stem rust greenhouse in Egypt and GRRC in Denmark. The geographical distribution of the most virulent races: TTKTT, TTKST, and TTKTK are found in Iran, Kenya, Rwanda, Tanzania, and Uganda), TTTSK is only found in Egypt, and TTKSK is found in Egypt, Iran, Kenya, Rwanda, Tanzania, and Uganda). The HCA and PCA-associated loading verified the substantial correlation between Kenya, Tanzania, Uganda, Rwanda, and Iran. Egypt, on the other hand, was adversely connected with the majority of the analyzed locations, including Eritrea, Spain, Ethiopia, Morocco, Italy, Poland, Kenya, Tanzania, and Uganda, or had no significant relationship, including Tunisia, Denmark, France, and Rwanda. Through this, it became clear that there is a relationship between the most virulent races (TTKSK, TTKTK, TTTSK, TTKST, TTKTT, TTRTF, and TTTTF) and the resistant monogenic lines (*Sr11*, *Sr31*, and *Sr24*) that fell into the same cluster through HCA and the PCA-associated loading.

Based on the North American naming system [28], isolates of Ug99 obtained in Kenya were identified as TTKS and were renamed TTKSK after adding a fifth set of differentials to the nomenclature system [20]. Given the gravity of this race, TTKSK (Ug99), it was vital to prove its existence using molecular markers in the majority of the nations where it was defined. The diagnostic PCR fragments indicate virulent TTKSK (Ug99) to *Sr31*, Clement (*Sr31*), and Brigardier (*Sr31*) as a fragment of 350 bp. This was in agreement with what was defined in the greenhouse (TTKSK) in Egypt and other countries based on the North American nomenclature [7,24].

Collectively, the findings of this study highlight the emergence of more aggressive races in Egypt, such as TTKSK, that break the resistance of wheat lines carrying *Sr31, Sr24, Sr36,* and *Sr38* resistance. These aggressive races also appeared in some other countries such as Iran, Kenya, Rwanda, Tanzania, and Uganda. The TTTKS race is discovered for the first time in Egypt. As a result, the findings caution against breaking the resistance of Egyptian and international types bearing these genes. Because they were prone to infection with this disease, particularly the fungal races, it is vital in breeding efforts for rusts, particularly stem rust, to look for more efficient resistance genes and to incorporate more than one gene in one variety.

More research is needed to determine the genetic basis of existing wheat stem rust resistance in Egyptian wheat breeding materials (genotypes) and other nations. It is critical to understand the stem rust resistance genes that are now deployed in wheat cultivars in order to establish which genes provide long-lasting protection and more durable resistance in these wheat cultivars. This understanding will help alert pathologists and breeders to wheat cultivars’ genetic vulnerability in addition to assisting us in developing disease-resistant varieties and how to trade these types with one another in order to ensure global food security.

## 5. Conclusions

Due to their migration to countries with the bulk of the stem rust resistance genes (*Sr*) and the complex virulence combinations they carry, the Ug99 group of wheat stem rust fungus races continue to pose a threat to the global supply of wheat. In Central West Asia (CWANA), 10 African nations, 10 European nations, and at least 14 stem rust races have been recognized. Four races—TTKSK, TTKST, TTKTK, and TTKTT—came from five African nations—Egypt, Kenya, Rwanda, Tanzania, and Uganda—as well as Iran—but none from Europe—in the Ug99 group. On the other hand, TTRTF, TKTTF, and TTTTF were not discovered in Egypt. Kenya, Tanzania, Uganda, Rwanda, and Iran were all closely associated with one another, according to a correlation analysis. Contrarily, Egypt had a negative correlation with the majority of the countries examined, including Eritrea, Spain, Ethiopia, Morocco, Italy, Poland, Kenya, Tanzania, and Uganda, or at least no significant correlation was found, including Tunisia, Denmark, France, and Rwanda. By using molecular markers, Ug99 (TTKSK) was validated in Egypt. Only in Egypt did the TTTSK race become known.

## Figures and Tables

**Table 1:** The list of wheat monogenic lines and varieties with *Sr* genes (*Sr’*s) used in this study.

No.	Name	Source ^a^	*Sr* Gene
1	ISr5-Ra	12 Aberdeen	*5*
2	CnS_T_monococcum	10 Aberdeen	*21*
3	Vernstein	11 Aberdeen	*9e*
4	ISr7b-Ra	10 Aberdeen	*7b*
5	ISr11-Ra	11GH	*11*
6	ISr6-Ra	12 Aberdeen	*6*
7	ISr8a-Ra	10 Aberdeen	*8a*
8	CnsSr 9g	10 Aberdeen	*9g*
9	W2691SrTt-1	11GH	*36*
10	W2691Sr9b	10 Aberdeen	*9b*
11	BtSr30Wst	11 Aberdeen	*30*
12	Combination	10 Aberdeen	*17*
13	ISr9a-Ra	10 Aberdeen	*9a*
14	ISr9d-Ra	10 Aberdeen	*9d*
15	W2691Sr10	10 Aberdeen	*10*
16	CnsSr Tmp	08 Aberdeen	*Tmp*
17	LcSr24Ag	08 Aberdeen	*24*
18	Sr31 (Benno)/6*LMPG-6 DK42	11 Aberdeen	*31*
19	VPM-1	13 Pullman	*38*
20	McNair 701	Griffey 2011	*MCN*
21	Kavkaz	-	*31*
22	Federation 4/Kavkaz	-	*31*
23	Brigardier	-	*31*
24	Clement	-	*31*
25	PBW343	-	*31*
26	Siouxland	06 NB	*24 + 31*
27	Sisson	12 Aberdeen	*36 + 31*
28	Roughrider	Hard red winter ^b^	*36, 6*
29	Fleming	Soft red winter	*6, 24, 36, 1RS-Am*
30	Chris	-	*7a, 12, 6*

^a^ Wheat lines with stem rust resistance genes (*Sr’s*) were obtained from the International Maize and Wheat Improvement Center (CIMMYT, Mexico). “Aberdeen” refers to the National Small Grain Collection (Aberdeen, ID, USA), whereas the “numbers” beside it refer to the year of deposit. ^b^ Hard Red Winter Wheat Variety.

**Table 2:** Stem rust samples preserved in Global Rust Reference Center (GRRC) in Denmark according to Hovmøller et al. [19].

Geographic Area	Country		2019			2020			Grand Total
			Dead	Recovered	Total	Dead	Recovered	Total	
Africa, CW Asia		Egypt	21	2	23	-	-	-	23
		Eritrea	7	12	19	-	--	-	19
		Ethiopia	-	4	4	-	-	-	4
		Iran	25	22	47	-	12	12	59
		Kenya	9	68	77	1	61	62	139
		Morocco	-	-	-	-	2	2	2
		Rwanda	-	13	13	-	-	-	13
		Tanzania	1	27	28	-	-	-	28
		Tunisia	-	-	-	-	8	8	8
		Uganda	-	-	-	8	37	45	45
Africa, CW Asia Total			63	148	211	9	120	129	340
Europe		Czech Republic	-	5	5	-	-	-	5
		Denmark	1	-	1	-	9	9	10
		France	-	-	-	-	1	1	1
		Germany	-	-	-	-	2	2	2
		Hungary	-	-	-		1	1	1
		Italy	4	60	64	1	54	55	120
		Poland	-	14	14	-	-	-	14
		Slovakia	-	14	14	-	-	-	14
		Spain	-	-	-	2	73	75	76
		Sweden	8	29	37	2	5	7	44
Europe Total			13	122	135	5	145	150	285
Grand Total			76	270	346	14	265	279	625

Recovered: the urediniospores of stem rust pathogen, *P. graminis* f. sp. *tritici* were recovered from the collected stem samples and successfully initiated infections of susceptible wheat seedlings; otherwise, it was considered “dead”.

**Table 3:** *Puccinia graminis* Pt-code for five sets of the 20 differential hosts.

		Infection Type Produced on Near Isogenic *Sr* Lines			
Pt-code ^a^	Host set 1:	*5*	*21*	*9e*	*7b*
	Host set 2:	*11*	*6*	*8a*	*9g*
	Host set 3:	*36*	*9b*	*30*	*17*
	Host set 4:	*9a*	*9d*	*10*	*Tmp*
	Host set 5:	*24*	*31*	*38*	*McN*
B		L	L	L	L
C		L	L	L	H
D		L	L	H	L
F		L	L	H	H
G		L	H	L	L
H		L	H	L	H
J		L	H	H	L
K		L	H	H	H
L		H	L	L	L
M		H	L	L	H
N		H	L	H	L
P		H	L	H	H
Q		H	H	L	L
R		H	H	L	H
S		H	H	H	L
T		H	H	H	H

^a^ *P. graminis* coding (Pt-code) for each isolate was described using uppercase letters based on the combination of responses from low infection type (L; resistant reaction type or avirulence) and high infection type (H; susceptible reaction type or virulence) plants. Pt-code consists of the designation for set 1 followed by that for set 2, etc. The name of the race is given by five alphabetic letters from B to T. Table adapted from Jin et al. [20].

**Table 4:** Disease severity and reaction type of 30 wheat monogenic lines and varieties with stem rust resistance genes (*Sr*) at the two governorates during the three growing seasons.

Differential Line (Variety)	Sr Gene	Kafrelsheikh			Sharqia		
		2020	2021	2022	2020	2021	2022
ISr5-Ra	*5*	40S ^a^	70S	70S	10S	30S	60S
CnS_T_monococcum	*21*	40S	70S	80S	20S	30S	50S
Vernstein	*9e*	40S	60S	80S	20S	40S	50S
ISr7b-Ra	*7b*	30S	50S	70S	20S	50S	60S
ISr11-Ra	*11*	5MS ^b^	30S	50S	TrMS	10S	60S
ISr6-Ra	*6*	20S	40S	90S	5S	5S	60S
ISr8a-Ra	*8a*	40S	60S	80S	10S	60S	50S
CnsSr 9g	*9g*	20S	40S	80S	10S	40S	50S
W2691SrTt-1	*36*	0	TrMR ^c^	10S	0	5S	50S
W2691Sr9b	*9b*	20S	40S	60S	10S	20S	50S
BtSr30Wst	*30*	30S	60S	30S	10S	10S	60S
Combination	*17*	40S	70S	40S	20S	30S	60S
ISr9a-Ra	*9a*	20S	40S	70S	10S	30S	50S
ISr9d-Ra	*9d*	40S	60S	70S	10S	20S	40S
W2691Sr10	*10*	30S	60S	60S	10S	30S	40S
CnsSr Tmp	*Tmp*	50S	70S	70S	40S	60S	60S
LcSr24Ag	*24*	TrR ^d^	5R	40MS-S	TrMS	TrS	60MS
Sr31 (Benno)/6*LMPG-6 DK42	*31*	TrR	5MR	40MR-MS	TrR	TrMS	30MS
VPM-1	*38*	0	TrMS	5MS-S	0	0	40S
McNair 701	*MCN*	40S	60S	70S	30S	70S	70S
Kavkaz	*31*	0	5MR	10MR	0	10MR-MS	10MR-MS
Federation 4/Kavkaz	*31*	0	5MR	5R-MR	0	0	10MR-MS
Brigardier	*31*	0	0	10R-MR	0	0	10MR-MS
Clement	*31*	0	TrMR	TrMS	0	0	10MR-MS
PBW343	*31*	0	0	10R-MR	0	TRMR	10MR-MS
Siouxland	*24 + 31*	0	TrR-MR	30MR-MS	0	0	10MR-MS
Sisson	*36 + 31*	0	0	20MR-MS	0	TrMR	10MR-MS
Roughrider	*Sr36,6*	0	10MS-S	5MR-MS	0	0	10MR-MS
Fleming	*Sr6,24,36,1RS-Am*	0	TrR	5R	0	0	10MR-MS
Chris	*Sr7a, Sr12,Sr6*	0	0	TrR	0	0	10MR-MS

Numbers refer to the disease severity (%) of the stem rust pathogen, *P. graminis* f. sp. *tritici*, recorded after the heading stage using the modified Cobb’s scale, adopted by Peterson et al. [16], whereas letters refer to the reaction/infection types, i.e., highly resistant (0), resistant (R), moderately resistant (MR), moderately susceptible (MS), and susceptible (S) were recorded according to Roelfs et al. [17]. Tr= Trace.

**Table 5:** Stem rust samples collected from Kafrelsheikh and Sharqia governorates during the three growing seasons.

Location	2020		2021		2022		Total	
	NS	NI	NS	NI	NS	NI	NS	NI
Kafrelsheikh	8	6	8	4	5	3	21	13
Sharqia	15	9	12	7	9	4	36	20
Total	23	15	20	11	14	7	57	33

NS = Number of samples; NI = Number of isolates.

**Table 6:** The number of isolates and frequency of stem rust races identified in Kafrelsheikh and Sharqia governorates during the three growing seasons.

Race	Seasons/Governorates/Number of Isolates and Frequency												Total	
	2020				2021				2022					
	Kafrelsheikh		Sharqia		Kafrelsheikh		Sharqia		Kafrelsheikh		Sharqia			
	NI*	FR**	NI	FR	NI	FR	NI	FR	NI	FR	NI	FR	NI	FR
PKSTC	1	16.6	1	11.1	1	25.0	1	14.3	-	-	-	-	4	12.1
RKTTH	-	-	2	22.2	-	-	1	14.3	-	-	-	-	3	9.1
TCKTC	-	-	1	11.1	-	-	-	-	1	33.3	-	-	2	6.1
TKKTF	3	50.0	2	22.2	2	50.0	2	28.6	-	-	-	-	9	27.3
TKTTC	1	16.6	1	11.1	1	25.0	1	14.3	-	-	-	-	4	12.1
TKTTH	-	-	1	11.1	-	-	-	-	-	-	1	25.0	2	6.1
TTKSK	1	16.6	1	11.1	-	-	1	14.3	1	33.3	2	50.0	6	18.2
TTTSK	-	-	-	-	-	-	1	14.3	1	33.3	1	25.0	3	9.1
Total	6		9		4		7		3		4		33	100.0

NI* = Number of isolates and FR** = Frequency (%).

**Table 7:** Virulence formula of 14 *Puccinia graminis* races on 20 stem rust differential lines in Egypt and other countries.

Sr Gene	Reaction Type													
	PKSTC	RKTTC	TCKTC	TKKTF	TKTTC	TKTTD	TTKSK	TTTSK	TTKST	TTKTK	TTKTT	TTRTF	TKTTF	TTTTF
*Sr5*	*+*	*+*	*+*	*+*	*+*	*+*	*+*	*+*	*+*	*+*	*+*	*+*	*+*	*+*
*Sr21*	*-*	*+*	*+*	*+*	*+*	*+*	*+*	*+*	*+*	*+*	*+*	*+*	*+*	*+*
*Sr9e*	*+*	*-*	*+*	*+*	*+*	*+*	*+*	*+*	*+*	*+*	*+*	*+*	*+*	*+*
*Sr7b*	*+*	*+*	*+*	*+*	*+*	*+*	*+*	*+*	*+*	*+*	*+*	*+*	*+*	*+*
*Sr11*	*-*	*-*	*-*	*-*	*-*	*-*	*+*	*+*	*+*	*+*	*+*	*+*	*-*	*+*
*Sr6*	*+*	*+*	*-*	*+*	*+*	*+*	*+*	*+*	*+*	*+*	*+*	*+*	*+*	*+*
*Sr8a*	*+*	*+*	*-*	*+*	*+*	*+*	*+*	*+*	*+*	*+*	*+*	*+*	*+*	*+*
*Sr9g*	*+*	*+*	*+*	*+*	*+*	*+*	*+*	*+*	*+*	*+*	*+*	*+*	*+*	*+*
*Sr36*	*+*	*+*	*-*	*-*	*+*	*+*	*-*	*+*	*-*	*-*	*-*	*+*	*+*	*+*
*Sr9b*	*+*	*+*	*+*	*+*	*+*	*+*	*+*	*+*	*+*	*+*	*+*	*-*	*+*	*+*
*Sr30*	*+*	*+*	*+*	*+*	*+*	*+*	*+*	*+*	*+*	*+*	*+*	*+*	*+*	*+*
*Sr17*	*-*	*+*	*+*	*+*	*+*	*+*	*+*	*+*	*+*	*+*	*+*	*+*	*+*	*+*
*Sr9a*	*+*	*+*	*+*	*+*	*+*	*+*	*+*	*+*	*+*	*+*	*+*	*+*	*+*	*+*
*Sr9d*	*+*	*+*	*+*	*+*	*+*	*+*	*+*	*+*	*+*	*+*	*+*	*+*	*+*	*+*
*Sr10*	*+*	*+*	*+*	*+*	*+*	*+*	*+*	*+*	*+*	*+*	*+*	*+*	*+*	*+*
*SrTmp*	*+*	*+*	*+*	*+*	*+*	*+*	*-*	*-*	*-*	*+*	*+*	*+*	*+*	*+*
*Sr24*	*-*	*-*	*-*	*-*	*-*	*-*	*-*	*-*	*+*	*-*	*+*	*-*	*-*	*-*
*Sr31*	*-*	*-*	*-*	*-*	*-*	*-*	*+*	*+*	*+*	*+*	*+*	*-*	*-*	*-*
*Sr38*	*-*	*-*	*-*	*+*	*-*	*+*	*+*	*+*	*+*	*+*	*+*	*+*	*+*	*+*
*SrMcN*	*+*	*+*	*+*	*+*	*+*	*+*	*+*	*+*	*+*	*+*	*+*	*+*	*+*	*+*
Total*	14	15	13	16	16	17	17	18	18	18	19	17	17	18

(+) Virulent; (-) Avirulent; Total* = Total number of broken resistance genes/race.

## Data Availability

The data collected and analyzed throughout the present research are available upon request.
